# Clinical significance of postoperative pulmonary complications in elderly patients with lung cancer

**DOI:** 10.1093/icvts/ivac153

**Published:** 2022-05-30

**Authors:** Satoru Okada, Masanori Shimomura, Shunta Ishihara, Satoshi Ikebe, Tatsuo Furuya, Masayoshi Inoue

**Affiliations:** Division of Thoracic Surgery, Department of Surgery, Graduate School of Medical Science, Kyoto Prefectural University of Medicine, Kyoto, Japan; Division of Thoracic Surgery, Department of Surgery, Graduate School of Medical Science, Kyoto Prefectural University of Medicine, Kyoto, Japan; Division of Thoracic Surgery, Department of Surgery, Graduate School of Medical Science, Kyoto Prefectural University of Medicine, Kyoto, Japan; Division of Thoracic Surgery, Department of Surgery, Graduate School of Medical Science, Kyoto Prefectural University of Medicine, Kyoto, Japan; Division of Thoracic Surgery, Department of Surgery, Graduate School of Medical Science, Kyoto Prefectural University of Medicine, Kyoto, Japan; Division of Thoracic Surgery, Department of Surgery, Graduate School of Medical Science, Kyoto Prefectural University of Medicine, Kyoto, Japan

**Keywords:** Lung cancer, Surgery, Elderly patients, Postoperative pulmonary complications, Prognostic nutritional index, Operative time, Prognosis

## Abstract

**OBJECTIVES:**

An increasing number of elderly patients with impaired immunity, malnutrition and comorbidities are considered surgical candidates. This study aimed to clarify the predictive factors and prognostic impact of postoperative pulmonary complications in elderly patients with lung cancer.

**METHODS:**

This retrospective study included 188 patients (≥75 years) who underwent complete anatomical lung resection for non-small cell lung cancer between 2005 and 2019. Postoperative pulmonary complications graded ≥II in the Clavien-Dindo classification, occurring within 30-day post-surgery were evaluated. Multivariate logistic regression analyses and Cox proportional hazard models were used to analyse predictors and prognostic impact of complications.

**RESULTS:**

Video-assisted thoracoscopic surgery was performed in 154 patients (81.9%). The 90-day mortality rate was 0.5%. Postoperative pulmonary complications including air leak, pneumonia, sputum retention, atelectasis, bronchopleural fistula and empyema occurred in 29 patients (15.4%). A lower prognostic nutritional index (<45) and longer operative time were independent predictive factors of pulmonary complications, with 33.3% of patients experiencing both factors. Following a median follow-up of 48 months, the 5-year overall and relapse-free survival rates were significantly worse in patients with pulmonary complications than in those without them (54.4% vs 81.5% and 41.2% vs 74.9%). Pulmonary complications were significantly associated with worse overall and relapse-free survival [hazard ratio (95% confidence interval): 1.97 (1.01–3.66), *P *=* *0.047 and 2.35 (1.28–4.12), *P *=* *0.007, respectively] along with pathologic stage and carcinoembryonic antigen levels.

**CONCLUSIONS:**

Postoperative pulmonary complications are associated with a lower prognostic nutritional index and prolonged operative time; the complications are independent adverse prognostic factors in elderly patients.

**Figure:**
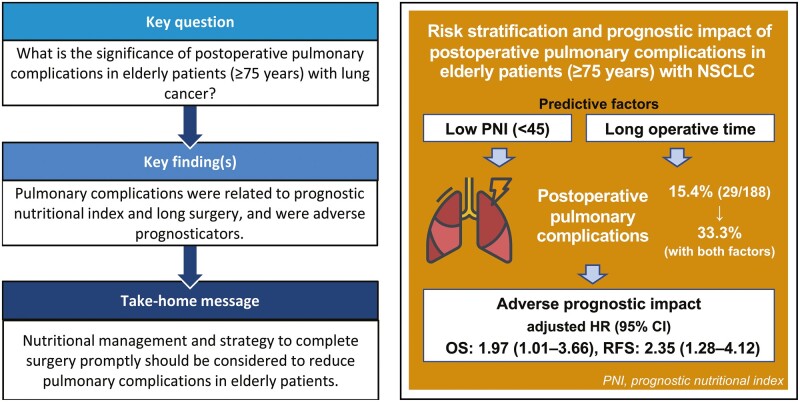

## INTRODUCTION

Lung cancer is the leading cause of cancer-related death worldwide [1], and surgical resection remains the standard treatment for localized diseases. Clinicians are increasingly dealing with elderly patients presenting with medically operable non-small cell lung cancer (NSCLC). In 2018, among the patients who underwent surgical resection for primary lung cancer in Japan, 58.7% and 13.6% were aged ≥70 and ≥80 years, respectively [2].

In many countries, elderly people are defined as a population with a chronological age of ≥65 years. However, the perception on the ‘elderly’ has been changing with the extension of healthy life expectancy. The phenomenon of ‘rejuvenation’ was characterized in a recent study analysing the physical (gait speed and grip strength) and psychological health of the elderly. Currently, the appearance of age-related changes in physical function has been delayed by 5–10 years compared to 10–20 years ago [3]. In Japan, the country with the oldest population, redefining the elderly population as individuals aged ≥75 years was proposed in 2017 [3]. Life expectancy has improved over time, and at 65 years old for women and men in 2019, it was reported as 24.6 and 19.8 years in Japan, 21.4 and 18.3 years in Germany and 20.8 and 18.2 years in the USA, respectively [4]. Therefore, it is important to consider offering opportunities of safe and curative treatment options for elderly patients (≥75 years) with lung cancer.

However, elderly people often present with impaired immunity, malnutrition, frailty or more comorbidities, which may negatively affect short- and long-term postsurgical outcomes. In addition, postoperative complications reportedly affect long-term surgical outcomes [5–7]. Although the present times have been regarded as the era of ageing, little is known about the clinical significance of postoperative complications and long-term survival of elderly patients after lung cancer surgery. Thus, this study aimed to identify clinically important postoperative complications and clarify the predictive factors and prognostic impact of these complications in elderly patients (≥75 years) who underwent lung cancer surgery.

## PATIENTS AND METHODS

### Ethical statement

The protocol for this study was approved by the Institutional Review Board of Kyoto Prefectural University of Medicine on 17 August 2020 (ERB-C-565-5). The requirement for informed consent was waived due to the retrospective nature of the study.

### Patients and clinicopathological characteristics

The data of 202 consecutive patients who underwent pulmonary anatomical resection (lobectomy or segmentectomy) for NSCLC at the Kyoto Prefectural University of Medicine between January 2005 and October 2019 were retrospectively reviewed. After excluding 14 patients who underwent incomplete resections, 188 patients were included in the final analysis.

Clinicopathological characteristics obtained from patient medical records were evaluated. As a biomarker for immunonutritional status, the prognostic nutritional index (PNI) was calculated as 10 × serum albumin level (g/dl) + 0.005 × total lymphocyte count (/mm^3^) using preoperative data, and the threshold was determined based on Onodera’s original cut-off [8, 9]; a PNI of <45 indicated malnutrition. The evaluated comorbidities included hypertension, ischaemic heart disease, cerebrovascular disease, chronic kidney disease (estimated glomerular filtration rate <60 ml/min/1.73 m^2^), obstructive pulmonary disease, interstitial pneumonia and diabetes mellitus. The postoperative staging was performed according to the seventh edition of the International Association for the Study of Lung Cancer TNM Classification [10].

### Surgical policy and postoperative complications

Operability was assessed based on electrocardiography, pulmonary function tests and arterial blood gas analysis. Echocardiography and/or a stair-climbing test were performed in patients with relatively low cardiopulmonary function. Video-assisted thoracoscopic surgery (VATS) was the standard approach to treatment; large tumours and nodal diseases were treated via open thoracotomy. Lobectomy was the standard mode of surgery; segmentectomy was an option for patients with small peripheral or ground-glass dominant tumours or deemed ineligible for lobectomy.

The severity of postoperative complications was defined according to the Clavien-Dindo classification [11]. Complications of grade ≥II that required pharmacological and/or surgical intervention were included in this study. Pulmonary complications included air leak, pneumonia, sputum/atelectasis, empyema and bronchopleural fistula. Air leak was defined as an air leakage lasting ≥7 days or requiring medical or surgical intervention to cease.

### Outcomes

The primary outcomes of this study were the frequency, predictive factors and prognostic impact of postoperative pulmonary complications in elderly patients with NSCLC.

### Statistical analyses

Comparisons among groups were performed using the Wilcoxon rank-sum test for continuous variables and the Chi-squared or Fisher’s exact test for categorical variables. The median values were used as the cut-off for categorical comparisons of smoking (pack-years), operative time and blood loss. To compare the association between nutritional factors and pulmonary complications, a receiver operating characteristic curve analysis was performed.

To identify independent predictive factors of pulmonary complications, multivariate logistic regression analyses were performed. Variables with *P *<* *0.05 in the univariate analysis were entered into the multivariate analysis and only variables with *P *<* *0.05 were included in a final model using forward and backward stepwise selection methods. For risk stratification of postoperative pulmonary complications, patients were divided into 4 groups based on the combination of significant factors they presented with.

Overall survival (OS) was defined as the time elapsed from the date of surgery to either that of death from any cause or of the last follow-up. Relapse-free survival (RFS) was defined as the time elapsed from the date of surgery to that of initial recurrence, death from any cause or the last follow-up. Survival curves were estimated using the Kaplan–Meier method and compared using the log-rank test. To identify independent prognostic factors, multivariate Cox proportional hazard models were performed using variables selected in the same manner as the multivariate logistic regression analyses.

Statistical significance was set at *P *<* *0.05. Statistical analyses were performed using the JMP software package (version 13.0; SAS Institute Inc., Cary, NC, USA).

## RESULTS

### Patients’ clinicopathological characteristics

The median age of the patients was 78 years [range: 75–92 years; interquartile range (IQR): 76, 81]. There were 69 patients aged ≥80 years (36.7%). The median preoperative PNI score was 48.5 (range: 30.8–60.3; IQR: 45.2, 51.6). VATS was performed in 154 patients (81.9%). During the median follow-up period of 48 months (range: 2–138 months, IQR: 28, 65), 34 cases of relapse and 47 cases of death were recorded. A summary of the clinicopathological characteristics of the patients is presented in Table 1. Compared with patients aged 75–79 years, those aged ≥80 years had a significantly higher incidence of comorbidity (at least one), hypertension or chronic kidney disease and a lower PNI (Supplementary Material, Table S1).

**Table 1:** Clinicopathological characteristics of 188 elderly patients (≥75 years) with non-small cell lung cancer

Characteristics	Median (IQR) or *N* (%)
Age (years)	78 (76, 81)
Sex	
Male	118 (62.8)
Female	70 (37.2)
BMI (kg/m^2^)	22.9 (20.5, 24.7)
Smoking history (yes)	122 (64.9)
Smoking (pack-years)	20 (0, 55)
%FEV1 (%)	107 (89, 124)
%VC (%)	100 (88.9,115)
Total protein level (g/dl)	6.9 (6.6, 7.2)
Albumin level (g/dl)	4.1 (3.9, 4.3)
Total lymphocyte count (×10^3^/mm^3^)	1.47 (1.13, 1.83)
Haemoglobin (g/dl)	12.8 (11.5, 13.8)
Prognostic nutritional index	48.5 (45.2, 51.6)
Comorbidity (with at least one)	131 (69.7)
Hypertension	105 (55.9)
Diabetes mellitus	43 (22.9)
Ischaemic heart disease	14 (7.5)
Cerebrovascular disease	19 (10.1)
Chronic kidney disease	55 (29.3)
COPD	58 (30.9)
Interstitial pneumonia	6 (3.2)
CEA level (>5 ng/ml)	60 (31.9)
Surgical approach	
VATS	154 (81.9)
Open thoracotomy	34 (18.1)
Mode of surgery	
Lobectomy	152 (80.9)
Segmentectomy	36 (19.1)
Bronchoplasty	3 (1.6)
Pleural adhesion	55 (29.3)
Operative time (min)	203 (165, 245)
Blood loss (g)	20 (4, 91)
Histology	
Adenocarcinoma	134 (71.3)
Non-adenocarcinoma	54 (28.7)
Pathologic stage	
I	144 (76.6)
II	29 (15.4)
III	15 (8.0)
Induction treatment	5 (2.7)
Adjuvant chemotherapy	59 (31.4)

BMI: body mass index; CEA: carcinoembryonic antigen; COPD: chronic obstructive pulmonary disease; IQR: interquartile range; NSCLC: non-small cell lung cancer; %FEV1: % predicted forced expiratory volume in 1 s; %VC: % predicted vital capacity; VATS: video-assisted thoracoscopic surgery.

### Postoperative complications

Postoperative complications (grade ≥II) occurred in 68 patients (36.2%). The most common complications were atrial fibrillation (*n* = 32; 17.0%), air leak (*n* = 17; 9.0%) and pneumonia (*n* = 9; 4.8%; Table 2). Postoperative pulmonary complications occurred in 29 patients (15.4%). Severe complications (grade ≥III) occurred in 21 patients (11.2%). There was no in-hospital mortality. Within 90 days of surgery, only 1 patient died due to the progression of other malignancies after discharge. The 30- and 90-day mortality rates were 0% and 0.5%, respectively.

**Table 2:** Postoperative complications

Outcomes	*N* (%)	Postoperative hospital stay (days)^a^
Complication (grade I) or none	120 (63.8)	9 (8, 12)
Complication (grade II)	47 (25.0)	13 (11, 16)
Complication (grade ≥III)	21 (11.2)	18 (13, 31)
Cardiovascular complications	32 (17.0)	13 (11, 16)
Atrial fibrillation	32 (17.0)	13 (11, 16)
Myocardial infarction	0	–
Brain infarction/haemorrhage	0	–
Pulmonary complications	29 (15.4)	17 (13, 28)
Air leak	17 (9.0)	16 (13, 22)
Pneumonia	9 (4.8)	19 (14, 30)
Atelectasis/Sputum	5 (2.7)	26 (11, 53)
Bronchopleural fistula	2 (1.0)	109 (99, 119)
Empyema	1 (0.5)	29 (29, 29)
Extrapulmonary infection^b^	8 (4.3)	16 (13, 28)
Bleeding	4 (2.1)	12 (10, 17)
Delirium	4 (2.1)	13 (9, 23)
Others	6 (1.6)	17 (14, 25)

a Presented as median (interquartile range).

b Surgical site infection, urinary tract infection and bacteraemia.

The median duration of postoperative hospital stay for all 188 patients and in the 68 patients with complications (grade ≥II) was 11 (IQR: 8, 14) and 14 (IQR: 11, 20) days, respectively. The duration gradually increased according to the severity of the complications (Table 2). Patients with pulmonary complications had a significantly longer hospital stay than those without them [∼7 days longer; 17 (IQR: 13, 28) vs 10 (IQR: 8, 12) days, *P *<* *0.001].

### Predictors of postoperative pulmonary complications

Postoperative pulmonary complications were significantly associated with preoperative poor nutritional status (Supplementary Material, Table S2). Among body mass index, total protein level, albumin level, total lymphocyte count, PNI and haemoglobin level, PNI had the highest predictive value and only PNI was significantly associated with pulmonary complications in both the continuous and categorical variable analyses. Therefore, considering multicollinearity, the PNI (<45) was used as a representative nutritional factor for further analyses.

Pulmonary complications were significantly associated with male sex, greater smoking pack-years, lower PNI, pleural adhesion, longer operative time, greater blood loss, open thoracotomy and advanced p-stage (Supplementary Material, Table S3). They also showed a non-significant trend towards obstructive pulmonary disease and restrictive pulmonary disorders (% predictive vital capacity <80%). Finally, there were no clear associations with other comorbidities.

**Figure 1:**
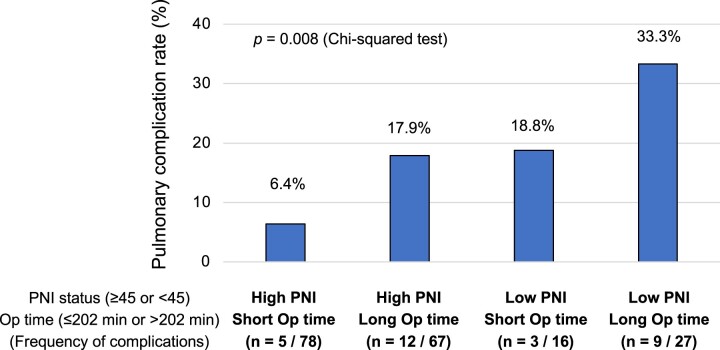
Frequency of postoperative pulmonary complications according to prognostic nutritional index and operative time in elderly patients (≥75 years) with NSCLC. NSCLC: non-small cell lung cancer; Op: operative; PNI: prognostic nutritional index.

Multivariate analysis revealed that a lower PNI and longer operative time were significant independent relevant predictors of postoperative pulmonary complications (Table 3). Operative time was significantly associated with male sex, smoking history, lower % predictive forced expiratory volume in 1 s, restrictive pulmonary disorders, lower PNI, open thoracotomy, pleural adhesion, greater blood loss, non-adenocarcinoma histology and advanced disease (Supplementary Material, Table S4). When patients were stratified into 4 groups according to PNI and operative time, the frequencies of pulmonary complications in patients with a higher PNI and shorter operative time and those with a lower PNI and longer operative time were 6.4% and 33.3%, respectively (*P *= 0.008; Fig. 1).

**Table 3:** Univariate and multivariate analysis for predicting postoperative pulmonary complications

Variables	Category	*N*	Pulmonary complications	*P*	Multivariate analysis	
					OR (95% CI)	*P*
Age (years)	<80	119	16 (13.5%)	0.32		
	≥80	69	13 (18.8%)			
Sex	Male	118	24 (20.3%)	**0.015**		
	Female	70	5 (7.1%)			
BMI (kg/m^2^)	<18.5	19	4 (21.1%)	0.43		
	≥18.5, <25	127	21 (16.5%)			
	>25	42	4 (9.5%)			
Smoking history	No	66	7 (10.6%)	0.18		
	Yes	122	22 (18.0%)			
Smoking (pack-year)	≤20	94	9 (9.6%)	**0.026**		
	>20	94	20 (21.3%)			
%FEV1 (%)	<80	28	6 (21.4%)	0.34		
	≥80	160	23 (14.4%)			
%VC (%)	<80	21	6 (28.6%)	0.08		
	≥80	167	23 (13.8%)			
Comorbidity	Hypertension	105	18 (17.1%)	0.46		
	Diabetes mellitus	43	7 (16.3%)	0.86		
	Ischaemic heart disease	14	2 (14.3%)	>0.99		
	Cerebrovascular disease	19	1 (5.3%)	0.32		
	Chronic kidney disease	55	10 (18.2%)	0.50		
	COPD	58	13 (22.4%)	0.08		
	Interstitial pneumonia	6	2 (33.3%)	0.23		
	None	57	7 (12.3%)	0.43		
PNI	<45	43	12 (27.9%)	**0.010**	2.57 (1.09–6.04)	**0.031**
	≥45	145	17 (11.7%)		Reference	
CEA level (ng/ml)	≤5	128	18 (14.1%)	0.45		
	>5	60	11 (18.3%)			
Approach	VATS	154	20 (13.0%)	**0.049**		
	Open thoracotomy	34	9 (26.5%)			
Mode of surgery	Lobectomy	152	25 (16.5%)	0.43		
	Segmentectomy	36	4 (11.1%)			
Pleural adhesion	Present	55	13 (23.6%)	**0.045**		
	Absent	133	16 (12.0%)			
Operative time (min)	≤202	94	8 (8.5%)	**0.009**	Reference	**0.023**
	>202	94	21 (22.3%)		2.79 (1.15–6.77)	
Blood loss (g)	≤20	98	9 (9.2%)	**0.013**		
	>20	90	20 (22.2%)			
Histology	Adenocarcinoma	134	19 (14.2%)	0.46		
	Non-adenocarcinoma	54	10 (18.5%)			
Pathologic stage	I	144	20 (13.9%)	0.29		
	≥II	44	9 (20.5%)			

Significant values are highlighted in boldface.

BMI: body mass index; CEA: carcinoembryonic antigen; CI: confidence interval; COPD: chronic obstructive pulmonary disease; OR: odds ratio; %FEV1: % predicted forced expiratory volume in 1 s; %VC: % predicted vital capacity; PNI: prognostic nutritional index; VATS: video-assisted thoracoscopic surgery.

### Long-term prognosis according to postoperative pulmonary complications

**Figure 2:**
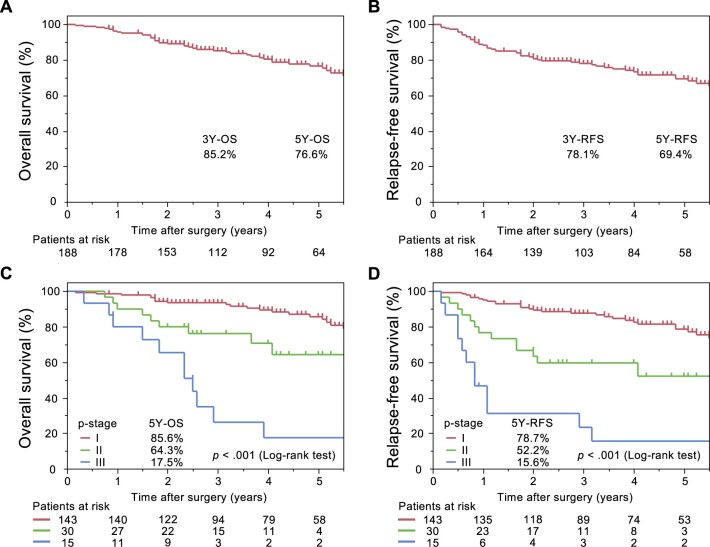
Overall and relapse-free survival in elderly patients (≥75 years) with completely resected NSCLC. NSCLC: non-small cell lung cancer; OS: overall survival; p-stage: pathologic stage; RFS: relapse-free survival.

The 5-year OS and RFS rates were 76.6% and 69.4%, respectively (Fig. 2A and B). The median survival time was 112 months for both the OS and RFS rates. Survival curves according to the pathologic stage were significantly different (Fig. 2C and D). The 5-year OS and RFS rates in patients aged ≥80 years were 72.2% and 63.0%, respectively (Supplementary Material, Fig. S1).

**Figure 3:**
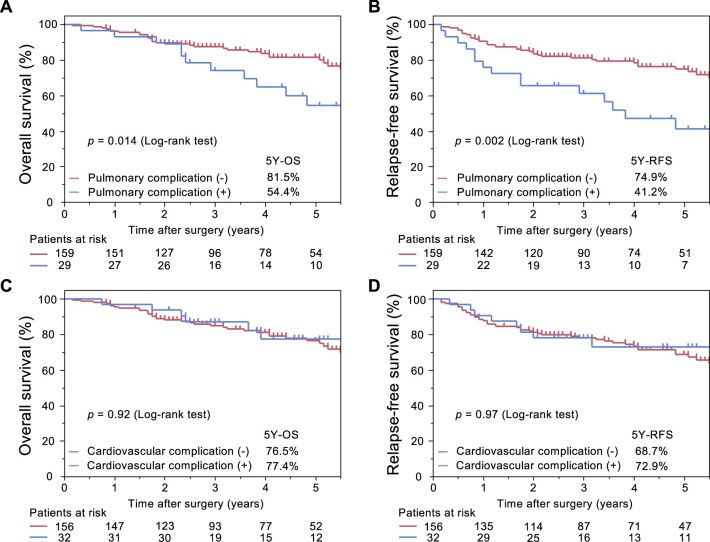
Overall and relapse-free survival in elderly patients (≥75 years) with completely resected NSCLC according to postoperative complications. NSCLC: non-small cell lung cancer; OS: overall survival; RFS: relapse-free survival.

Patients with postoperative pulmonary complications experienced a significantly higher incidence of recurrence and death than those without them (Table 4). The 5-year OS rates of patients with and those without pulmonary complications were 54.4% and 81.5%, respectively, and the corresponding RFS rates were 41.2% and 74.9%, respectively (Fig. 3A and B). Both OS and RFS rates were significantly worse in patients with pulmonary complications than in those without them (OS: *P *=* *0.014; RFS: *P *=* *0.002). Meanwhile, no significant difference was observed in both OS and RFS according to the occurrence of postoperative cardiovascular complications (Fig. 3C and D).

**Table 4:** Comparison of postoperative outcome according to postoperative pulmonary complications

Postoperative outcome	Pulmonary complications^a^		*P*
	No (*N* = 159)	Yes (*N* = 29)	
Postoperative stay (days)	10 (8, 12)	17 (13, 28)	**<0.001**
30-day mortality	0 (0)	0 (0)	>0.99
90-day mortality	1 (0.6)	0 (0)	>0.99
Recurrence	24 (15.1)	10 (34.5)	**0.013**
Death	33 (20.8)	14 (48.3)	**0.010**
Death according to cause			0.75
Relapse	16 (10.0)	7 (25.0)	
Other cancer	5 (3.1)	1 (3.6)	
Other than cancer	10 (6.3)	5 (17.9)	

Significant values are highlighted in boldface.

a Presented as number (%) or median (interquartile range).

The cause of death did not differ significantly between patients with and those without pulmonary complications (Table 4). However, the incidence of death due to relapse was more than twice as high in patients with pulmonary complications than in those without them, and that due to non-cancer-related death was almost 3 times higher. The proportion of patients who received adjuvant chemotherapy did not differ between patients with and those without pulmonary complications (41.4% vs 29.6%, *P *=* *0.21; Supplementary Material, Table S3).

### Prognostic significance of postoperative pulmonary complications for survival

In univariate analysis, pulmonary complications, male sex, smoking, interstitial pneumonia, carcinoembryonic antigen level, open thoracotomy, operative time, non-adenocarcinoma histology and p-stage were significantly associated with poor prognosis (Supplementary Material, Table S5). Multivariate analyses revealed that postoperative pulmonary complications were independent poor prognostic factors for both OS and RFS rates, along with carcinoembryonic antigen level (5 ng/ml) and p-stage (≥II; Table 5).

**Table 5:** Multivariable Cox proportional hazard model analysis for overall and relapse-free survival

Variables	Overall survival		Relapse-free survival	
	HR (95% CI)	*P*	HR (95% CI)	*P*
Postoperative pulmonary complications	1.97 (1.01–3.66)	**0.047**	2.35 (1.28–4.12)	**0.007**
CEA level (>5 ng/ml)	2.40 (1.33–4.32)	**0.004**	2.79 (1.64–4.73)	**<0.001**
Pathologic stage (≥II)	4.13 (2.24–7.54)	**<0.001**	4.30 (2.50–7.32)	**<0.001**

Significant values are highlighted in boldface.

CEA: carcinoembryonic antigen; CI: confidence interval; HR: hazard ratio.

## DISCUSSION

In this study, a lower PNI and a longer operative time were significantly associated with postoperative pulmonary complications in elderly patients with NSCLC. Pulmonary complications were the most frequent postoperative complications that prolonged hospital stay. Moreover, postoperative pulmonary complications had a significant adverse impact on both OS and RFS; thus, we regarded them as the most important complication in the management of elderly patients with NSCLC. Here, we first report the predictive factors and prognostic impact of postoperative pulmonary complications in elderly patients with NSCLC.

A low PNI represents poor immunonutritional status and is associated with impaired tissue healing and weak immunity, thereby leading to the development of prolonged air leak, pneumonia and other infectious complications [8, 9, 12]. Poor nutrition, characterized by low albumin levels, was also an independent predictor of postoperative complications in octogenarian patients in a Japanese nationwide study [13]. Although a lower PNI is reportedly associated with postoperative complications in patients of all ages [9, 12, 14], our data emphasize the importance of risk assessment using the PNI as an indicator of immunonutritional status in elderly patients with NSCLC. The presence of pneumonia should be considered because elderly patients are more likely to develop pneumonia than younger patients due to their poor oral hygiene, weakened cough reflex, reduced swallowing ability and poor immunonutritional status. Perioperative nutritional support and enhanced recovery could help [15], although limited evidence exists to support this notion.

The association between a longer operative time and postoperative complications found in this study was identical to that of Shiono’s study of elderly patients [16]. Prolonged operative time was associated with difficult and/or open surgeries with greater invasiveness during which surgeons may have dealt with advanced disease, pleural adhesion or damaged lung parenchyma (Supplementary Material, Table S4). Thus, prolonged operative time could be an indirect indicator of surgical invasiveness. Berry *et al.* [17] reported higher morbidity and mortality rates in patients aged >75 years who underwent open thoracotomy than in those who underwent VATS, arguing that the difference was due to the greater invasiveness to the chest wall by open thoracotomy. When our cohort was stratified according to surgical approach and PNI status, the pulmonary complication rate was 40.0% in those who underwent open thoracotomy and presented with a lower PNI, and 11.1% in those who underwent VATS and presented with a higher PNI (Supplementary Material, Fig. S2), showing a similar trend to that of Fig. 1. A longer operative time involves greater invasiveness and patient burden, which cause more inflammation and impair the healing process. In addition, a longer duration of general anaesthesia using one-lung ventilation may lead to incomplete awakening from anaesthesia and subsequent lung expansion. Furthermore, we previously reported that older patients or those with longer one-lung ventilation duration tended to have insufficient oxygenation after lung surgery [18]. These findings suggest that pulmonary complication rates in elderly patients would increase after surgeries requiring long operative time and/or open thoracotomy; thus, clinicians should consider adopting minimally invasive strategies and surgical approaches that can be achieved without an excessive duration.

Regarding long-term outcomes in elderly patients, the 5-year OS and RFS rates of 76.6% and 69.4% in our study were similar to those observed in recent reports, ranging from 40.8% to 60% and 53.4% to 56%, respectively [19–21]. Morbidity and mortality rates reportedly ranged from 20% to 65.9% and 0% to 6.2% [13, 15, 19–22], whereas morbidity (grade ≥II) and 90-day mortality rates in our study were 36.2% and 0.5%, respectively. Our data suggest that surgical intervention can be a safe treatment option for selected elderly patients.

It is not easy to clarify whether postoperative complications are causative factors for poor prognosis. Pulmonary complications were also associated with patients with poor prognostic factors (male sex, smoking, open thoracotomy and advanced stage). However, even after adjusting these confounding factors in multivariate analyses, pulmonary complications remained independent poor prognostic factors. This finding was in line with that of a previous study that included both young and elderly patients [7].

Although there were some concerns that postoperative complications might have compromised the prognosis by discouraging the use of adjuvant chemotherapy, there was no difference in the employment of adjuvant chemotherapy between patients with and those without pulmonary complications (Supplementary Material, Table S3). Among the frequently observed complications, we mainly treated air leaks with chemical pleurodesis using OK432 (Picibanil; Chugai Pharmaceutical Co., Tokyo, Japan) or talc. Pleurodesis impairs postoperative pulmonary function [23], which may negatively affect postoperative activity of daily life. Moreover, both pneumonia and pleurodesis can cause strong systemic inflammation, which might determine progression of the remaining cancer cells by modulation of the tumour microenvironment and muscle strength wasting due to protein catabolism [24, 25]. This could lead to increased oncological recurrence/death and non-oncological death. We also previously reported an adverse prognostic impact of inflammation characterized by an increase in postoperative C-reactive protein levels [26]. Taken together, poor OS and RFS could be partly explained by the adverse effects of inflammation induced by pulmonary complications and its treatment.

### Limitations

This study has some limitations that are related to the single-institution retrospective study design, selection bias, long period of evaluation and a relatively small sample size. Caution must be taken in generalizing the findings of this study because there may be some differences in the average lung function, body mass index and life expectancy between Western countries and Japan. Thus, external validation using extensive multi-institutional data in other regions is required to assess our results. However, in addition to the adaptability of using a generalized definition of complications, this study included detailed information such as comorbidity and nutritional factors, which most previous studies lacked. Therefore, our results may aid clinicians in carefully selecting a suitable surgical option for elderly patients and planning minimally invasive strategies and perioperative management.

## CONCLUSION

In conclusion, postoperative pulmonary complications have negative long-term prognostic impacts after anatomical lung resection in elderly patients with NSCLC. A lower preoperative PNI and prolonged operative time are independently associated with pulmonary complications. Nutritional management and minimally invasive strategy to complete surgery promptly should be considered to reduce pulmonary complications in elderly patients.

## SUPPLEMENTARY MATERIAL

Supplementary material is available at *ICVTS* online.

## Supplementary Material

ivac153_Supplementary_Data

## Data availability

Data available on request.

## ABBREVIATIONS

IQR: Interquartile range
NSCLC: Non-small cell lung cancer
OS: Overall survival
PNI: Prognostic nutritional index
RFS: Relapse-free survival
VATS: Video-assisted thoracoscopic surgery
