# Supplementary material for: Clinical significance of postoperative pulmonary complications in elderly patients with lung cancer
Source: Interact Cardiovasc Thorac Surg. 2022 May 30;35(2):ivac153. doi: 10.1093/icvts/ivac153 (PMC9297523; doi:10.1093/icvts/ivac153)
Supplement: ivac153_Supplementary_Data [file ivac153_supplementary_data.zip › Supplementary figure_R1.pdf]

Supplementary Figure S1. Overall and relapse-free survival in elderly patients (≥80 years old) with completely resected NSCLC

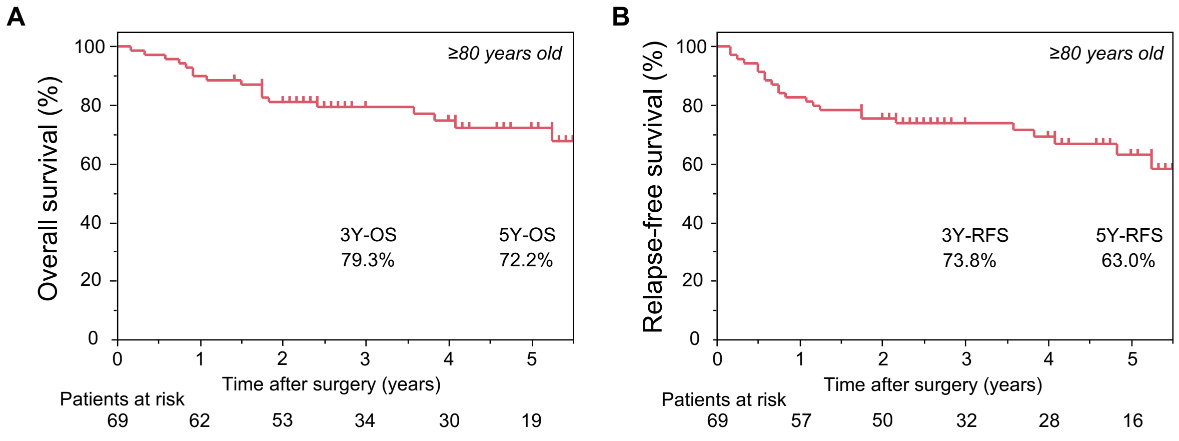

NSCLC, non-small cell lung cancer; *OS*, overall survival; *RFS*, relapse-free survival

Supplementary Figure S2. Frequency of postoperative pulmonary complications according to prognostic nutritional index and surgical approach in elderly patients (≥75 years old) with NSCLC

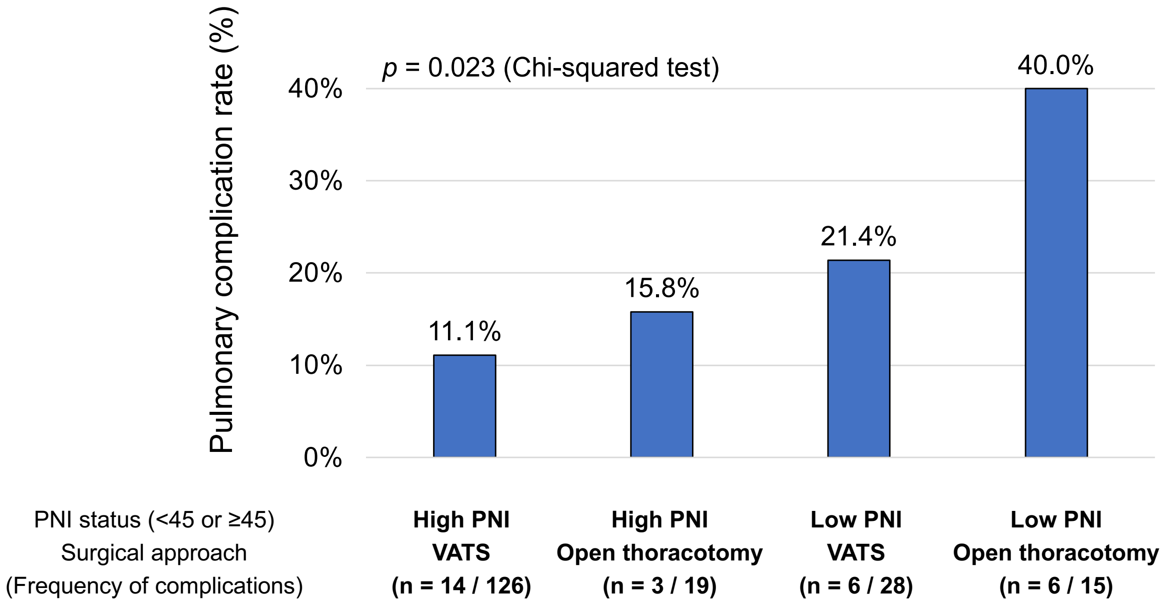

NSCLC, non-small cell lung cancer; *PNI*, prognostic nutritional index: *VATS*, video-assisted thoracoscopic surgery
