# Supplementary material for: Clinical significance of postoperative pulmonary complications in elderly patients with lung cancer
Source: Interact Cardiovasc Thorac Surg. 2022 May 30;35(2):ivac153. doi: 10.1093/icvts/ivac153 (PMC9297523; doi:10.1093/icvts/ivac153)
Supplement: ivac153_Supplementary_Data [file ivac153_supplementary_data.zip › Supplementary table_R1.pdf]

Supplementary Table S1. Comparison of patient characteristics between patients with NSCLC aged 75–79 years and those aged ≥80 years

Variables	Age (years)		*P*
	75–79 (N=119)	≥80 (N=69)	
Age (years)	77 (76, 78)	82 (81, 83)	-
Sex (Male)	73 (61.3)	45 (65.2)	.60 [b]
BMI (kg/m^2)	22.9 (20.5, 24.7)	22.7 (20.5, 24.8)	.92 [b]
BMI (kg/m^2)			.98 [b]
<18.5	12 (10.1)	7 (10.1)	
≥18.5, <25	81 (68.1)	21 (66.7)	
≥25	26 (21.9)	4 (23.2)	
Smoking history (yes)	76 (63.9)	46 (66.7)	.70 [b]
Smoking (pack-year)	20 (0, 55)	22.5 (0, 54)	.99 [a]
%FEV1 (<80%)	14 (11.8)	14 (20.3)	.11 [b]
%VC (<80%)	13 (10.9)	8 (11.6)	.89 [b]
Comorbidity (with at least one)	76 (63.9)	55 (79.7)	**.023** [b]
Hypertension	59 (49.6)	46 (66.7)	**.023** [b]
Diabetes mellitus	26 (21.9)	17 (24.6)	.66 [b]
Ischemic heart disease	7 (5.9)	7 (10.1)	.28 [b]
Cerebrovascular disease	11 (9.2)	8 (11.6)	.61 [b]
Chronic kidney disease	24 (20.2)	31 (44.9)	**<.001** [b]
COPD	35 (29.4)	23 (33.3)	.58 [b]
Interstitial pneumonia	4 (3.4)	2 (2.9)	>.99 [c]
PNI	48.8 (46, 51.9)	47.4 (42.8, 50.7)	**.009** [a]
PNI (<45)	20 (16.8)	23 (33.3)	**.009** [b]
CEA level (>5 ng/mL)	33 (27.7)	27 (39.1)	.11 [b]
Open thoracotomy	23 (19.3)	11 (15.9)	.56 [b]
Lobectomy	99 (83.2)	53 (76.8)	.28 [b]
Operative time (min)	204 (164, 243)	201 (166, 249)	.94 [a]
Blood loss (g)	20 (5, 85)	20 (3, 100)	.70 [a]
Histology			.35 [b]
Adenocarcinoma	82 (68.9)	52 (75.4)	
Non-adenocarcinoma	37 (31.1)	17 (24.6)	
Pathologic stage (≥II)	23 (19.3)	22 (31.9)	.05 [b]

Pathologic stage			.15 [b]
I	96 (80.7)	47 (68.1)	
II	15 (12.6)	15 (21.8)	
III	8 (6.7)	7 (10.1)	
Adjuvant chemotherapy	40 (33.6)	19 (27.5)	.39 [b]
Adjuvant chemotherapy			.15 [b]
Platinum-based	9 (7.6)	2 (2.9)	
Others	31 (26.1)	17 (24.6)	
None	96 (80.7)	47 (68.1)	
Adjuvant chemotherapy *	11 (47.8)	9 (40.9)	.64 [b]
Adjuvant chemotherapy *			.09 [b]
Platinum-based	8 (34.8)	2 (9.1)	
Others	3 (13.0)	6 (27.3)	
None	12 (52.2)	14 (63.6)	

[a] Wilcoxon rank-sum test, [b] Chi-squared test, [c] Fisher's exact test

* Analysis in patients with pathologic stage ≥II

Data are presented as median (interquartile range) or number (%).

BMI, body mass index; *CEA*, carcinoembryonic antigen; *COPD*, chronic obstructive pulmonary disease; *NSCLC,* non-small cell lung cancer; *%FEV1*, percent predicted forced expiratory volume in one second; *%VC*, percent predicted vital capacity; *PNI*, prognostic nutritional index

Supplementary Table S2. Relationship among nutritional factors and postoperative pulmonary complications in elderly patients (≥75 years old) with NSCLC

Nutritional factor (continuous variable)	Pulmonary complications			Logistic regression analysis		ROC curve analysis	
	No (N=169)	Yes (N=29)	*P*	OR (95% CI)	*P*	AUC	*P*
BMI (kg/m^2)	22.9 (20.6, 24,7)	22.5 (19.4, 24,5)	.15 [a]	0.90 (0.79–1.03)	.11	0.584	.11
TP level (g/dL)	6.9 (6.7, 7.2)	6.8 (6.6, 7.4)	.47 [a]	0.69 (0.31–1.55)	.37	0.542	.37
Alb level (g/dL)	4.1 (3.9, 4.3)	4.0 (3.6, 4.2)	.15 [a]	0.43 (0.16–1.11)	.09	0.583	.08
TLC (× 10^3/mm^3)	1.52 (1.19, 1.84)	1.30 (0.93, 1.77)	.10 [a]	0.57 (0.26–1.25)	.15	0.595	.16
PNI	48.7 (45.6, 51.7)	46.6 (42.4, 51.2)	.09 [a]	0.91 (0.85–0.99)	**.028**	0.599	**.029**
Hb level (g/dL)	12.8 (11.6, 13.9)	12.8 (11.0, 13.7)	.37 [a]	0.90 (0.71–1.14)	.38	0.552	.38

Nutritional factor (categorical variable)	N	Pulmonary complications			Logistic regression analysis	
		No (N=169)	Yes (N=29)	*P*	OR (95% CI)	*P*
BMI (<18.5 kg/m^2)	19	15 (9.4)	4 (13.8)	.50 [c]	1.54 (0.47–5.01)	.45
TP level (<6.5 g/dL)	24	18 (11.3)	6 (20.7)	.16 [b]	2.04 (0.73–5.69)	.17
Alb level (<3.5 g/dL)	13	8 (5.0)	5 (17.2)	**.017** [b]	3.93 (1.19–13.0)	**.025**
TLC (<1.0 × 10^3/mm^3)	29	20 (12.6)	9 (31.0)	**.011** [b]	3.13 (1.25–7.81)	**.015**
PNI (<45)	43	31 (19.5)	12 (41.4)	**.010** [b]	2.91 (1.26–6.73)	**.012**
Hb level (<12 g/dL)	64	52 (32.7)	12 (41.4)	.37 [b]	1.45 (0.65–3.26)	.37

[a] Wilcoxon rank-sum test, [b] Chi-squared test, [c] Fisher's exact test

Data are presented as medians (interquartile ranges) or numbers (%).

Alb, albumin; *AUC*, area under the curve; *BMI*, body mass index; *CI*, confidence interval; *Hb*, haemoglobin; *NSCLC,* non-small cell lung cancer; *OR*, odds ratio; *PNI*, prognostic nutritional index; *ROC*, receiver operating characteristic; *TLC*, total lymphocyte count; *TP*, total protein

Supplementary Table S3. Comparison of patient characteristics according to postoperative pulmonary complications

Variables	Pulmonary complications		*P*
	No (N=159)	Yes (N=29)	
Age (years)	78 (76, 81)	79 (77, 82)	.28 [a]
Sex (Male)	94 (59.1)	24 (82.8)	**.015** [a]
BMI (kg/m^2)	22.9 (20.6, 24.7)	22.5 (19.4, 24.5)	.15 [a]
BMI (kg/m^2)			.43 [b]
<18.5	15 (9.4)	4 (13.8)	
≥18.5, <25	106 (66.7)	21 (72.4)	
≥25	38 (23.9)	4 (13.8)	
Smoking history (yes)	100 (62.9)	22 (75.9)	.18 [b]
Smoking (pack-year)	20 (0, 50)	50 (5, 70)	**.019** [a]
Smoking (>20 pack-year)	74 (46.5)	20 (69.0)	**.026** [b]
%FEV1 (<80%)	22 (13.8)	6 (20.7)	.34 [b]
%VC (<80%)	15 (9.4)	6 (20.7)	.08 [b]
Comorbidity (with at least one)	133 (83.7)	27 (93.1)	.26 [c]
Hypertension	87 (54.7)	18 (62.1)	.46 [b]
Diabetes mellitus	36 (22.6)	7 (24.1)	.86 [b]
Ischemic heart disease	12 (7.6)	2 (6.9)	>.99 [c]
Cerebrovascular disease	18 (11.3)	1 (3.5)	.31 [c]
Chronic kidney disease	45 (28.3)	10 (34.5)	.50 [b]
COPD	45 (28.3)	13 (44.8)	.08 [b]
Interstitial pneumonia	4 (2.5)	2 (6.9)	.23 [c]
PNI	48.7 (45.6, 51.7)	46.6 (42.4, 51.2)	.09 [a]
PNI (<45)	31 (19.5)	12 (41.4)	**.001** [b]
CEA (>5 ng/mL)	49 (30.8)	11 (37.9)	.45 [b]
Open thoracotomy	25 (15.7)	9 (31.0)	**.049** [b]
Lobectomy	127 (79.9)	25 (86.2)	.61 [c]
Operative time (min)	194 (161, 241)	220 (184, 283)	**.016** [a]
Operative time (min)			**.043** [b]
≤Q_1 : –164	43 (27.1)	3 (10.4)	
>Q_1, ≤Q_2 : 165–202	43 (27.1)	5 (17.2)	
>Q_2, ≤Q_3 : 203–245	39 (24.5)	9 (31.0)	
>Q_3 : 246–	34 (21.3)	12 (41.4)	

Blood loss (g)	17 (3, 75)	50 (15, 176)	**.013** [a]
Blood loss (g)			.08 [b]
≤Q_1 : –4	44 (27.7)	3 (10.3)	
>Q_1, ≤Q_2 : 5–20	45 (28.3)	6 (20.7)	
>Q_2, ≤Q_3 : 21–91	34 (21.4)	9 (31.0)	
>Q_3 : 92–	36 (22.6)	11 (37.9)	
Histology			.46 [b]
Adenocarcinoma	115 (72.3)	19 (65.5)	
Non-adenocarcinoma	44 (27.7)	10 (34.5)	
Pathologic stage (≥II)	36 (22.0)	9 (31.0)	.33 [b]
Pathologic stage			**.020** [b]
I	123 (77.4)	20 (69.0)	
II	27 (17.0)	3 (10.3)	
III	9 (5.7)	6 (20.7)	
Adjuvant chemotherapy	47 (29.6)	12 (41.4)	.21 [b]
Adjuvant chemotherapy			.35 [b]
Platinum-based	8 (5.0)	3 (10.3)	
Others	30 (24.5)	9 (31.0)	
None	112 (70.4)	17 (58.6)	
Adjuvant chemotherapy [*]	16 (44.4)	4 (44.4)	>.99 [c]
Adjuvant chemotherapy [*]			.98 [b]
Platinum-based	8 (22.2)	2 (22.2)	
Others	7 (19.5)	2 (22.2)	
None	21 (58.3)	14 (55.6)	

[a] Wilcoxon rank-sum test, [b] Chi-squared test, [c] Fisher's exact test

[*] Analysis in patients with pathologic stage ≥II

Data are presented as median (interquartile range) or number (%).

BMI, body mass index; *CEA*, carcinoembryonic antigen; *COPD*, chronic obstructive pulmonary disease; *%FEV1*, percent predicted forced expiratory volume in one second; *%VC*, percent predicted vital capacity; *PNI*, prognostic nutritional index; *Q,* quartile

Supplementary Table S4. Associations between patient characteristics and operative time

Variables	Category	N	Operative time (min)	*P*
Age (years)	<80	119	204 (164, 243)	.94 [a]
	≥80	69	201 (166, 249)	
Sex	Male	118	220 (170, 267)	**<.001** [a]
	Female	70	185 (158, 217)	
BMI (kg/m^2)	<18.5	19	178 (137, 260)	.22 [a]
	≥18.5, <25	127	201 (164, 240)	
	>25	42	217 (180, 258)	
Smoking history	No	66	190 (163, 221)	**.038** [a]
	Yes	122	211 (168, 261)	
Smoking (pack-year)	≤20	94	194 (163, 229)	.09 [a]
	>20	94	211 (168, 261)	
%FEV1 (%)	<80	28	251 (205, 301)	**<.001** [a]
	≥80	160	194 (159, 238)	
%VC (%)	<80	21	232 (169, 296)	**.041** [a]
	≥80	167	197 (164, 241)	
Comorbidity	Hypertension	105	204 (166, 257)	.46 [a]
	Diabetes mellitus	43	196 (167, 257)	.86 [a]
	Ischemic heart disease	14	191 (167, 234)	.64 [a]
	Cerebrovascular disease	19	229 (170, 264)	.17 [a]
	Chronic kidney disease	55	194 (161, 241)	.73 [a]
	COPD	58	212 (177, 258)	.06 [a]
	Interstitial pneumonia	6	237 (198, 295)	.17 [a]
	None	57	185 (160, 230)	.10 [a]
PNI	<45	43	222 (180, 286)	**.013** [a]
	≥45	145	196 (159, 239)	
CEA level (ng/mL)	≤5	128	197 (164, 238)	.15 [a]
	>5	60	226 (166, 262)	
Approach	VATS	154	190 (158, 230)	**<.001** [a]

	Open thoracotomy	34	267 (212, 309)	
Mode of surgery	Lobectomy	152	205 (165, 249)	.12 [a]
	Segmentectomy	36	179 (162, 230)	
Pleural adhesion	Present	55	217 (170, 274)	**.036** [a]
	Absent	133	194 (159, 240)	
Blood loss (g)	$\leq Q_1$: –4	47	174 (147, 190)	**<.001** [a]
	$>Q_1, \leq Q_2$: 5–20	51	170 (145, 207)	
	$>Q_2, \leq Q_3$: 21–91	43	227 (187, 264)	
	$>Q_3$: 92–	47	257 (229, 307)	
Histology	Adenocarcinoma	134	188 (158, 229)	**<.001** [a]
	Non-adenocarcinoma	54	239 (189, 289)	
Pathologic stage	I	143	187 (157, 230)	**<.001** [a]
	II	30	242 (196, 297)	
	III	15	266 (226, 314)	

[a] Wilcoxon rank-sum test

Data are presented as median (interquartile range) or number.

BMI, body mass index; *CEA*, carcinoembryonic antigen; *COPD*, chronic obstructive pulmonary disease; *OR*, odds ratio; *%FEV1*, percent predicted forced expiratory volume in one second; *%VC*, percent predicted vital capacity; *PNI*, prognostic nutritional; *Q,* quartile; *VATS*, video-assisted thoracoscopic surgery

Supplementary Table S5. Univariate Cox proportional hazard model analysis of pulmonary complications and potential confounding factors for overall survival and relapse-free survival

Variables	Overall survival		Relapse-free survival	
	HR (95% CI)	*P*	HR (95% CI)	*P*
Pulmonary complications	2.16 (1.12–3.95)	**.024**	2.39 (1.31–4.17)	**.006**
Age (≥80 years)	1.46 (0.80–2.60)	.21	1.46 (0.86–2.46)	.16
Sex (Male)	2.83 (1.43–6.23)	**.002**	2.56 (1.40–5.05)	**.002**
BMI (<18.5 kg/m^2)	1.56 (0.59–3.45)	.34	1.16 (0.44–2.51)	.74
Smoking history (Yes)	1.70 (0.91–3.42)	.10	1.88 (1.06–3.55)	**.031**
Smoking (>20 pack year)	2.14 (1.18–4.01)	**.011**	1.65 (0.98–2.83)	.06
%FEV1 (<80%)	1.75 (0.75–3.62)	.18	1.62 (0.77–3.09)	.19
%VC (<80%)	1.94 (0.84–3.95)	.12	1.65 (0.75–3.19)	.20
Hypertension	1.28 (0.71–2.35)	.41	1.49 (0.88–2.59)	.14
Diabetes mellitus	1.10 (0.53–2.08)	.79	1.15 (0.61–2.04)	.66
Ischemic heart disease	1.17 (0.35–2.91)	.77	0.90 (0.27–2.21)	.84
Cerebrovascular disease	0.77 (0.23–1.90)	.61	0.77 (0.27–1.75)	.57
Chronic kidney disease	1.14 (0.59–2.09)	.69	1.07 (0.59–1.85)	.82
COPD	1.57 (0.84–2.83)	.15	1.38 (0.79–2.35)	.25
Interstitial pneumonia	3.35 (1.00–8.34)	**.049**	3.46 (1.04–8.56)	**.044**
PNI (<45)	1.91 (0.93–3.67)	.08	1.51 (0.79–2.72)	.20
CEA level (>5 ng/mL)	2.55 (1.42–4.54)	**.002**	2.83 (1.68–4.76)	**<.001**
Open thoracotomy	1.92 (0.98–3.56)	.06	2.19 (1.21–3.79)	**.011**
Lobectomy	1.62 (0.74–4.25)	.24	1.18 (0.62–2.47)	.63
Op time (>202 min)	2.11 (1.17–3.93)	**.012**	1.88 (1.11–3.24)	**.018**
Blood loss (>20 g)	1.58 (0.89–2.87)	.12	1.45 (0.86–2.46)	.16
Pleural adhesion (Present)	1.27 (0.66–2.30)	0.46	1.02 (0.57–1.77)	0.93
Non-adenocarcinoma	2.22 (1.23–3.94)	**.009**	2.12 (1.24–3.57)	**.006**
Pathologic stage (≥II)	3.99 (2.20–7.16)	**<.001**	4.29 (2.52–7.24)	**<.001**
Adjuvant chemotherapy	1.18 (0.65–2.11)	.58	1.57 (0.92–2.65)	.10

BMI, body mass index; *CEA*, carcinoembryonic antigen; *CI*, confidence interval; *COPD*, chronic obstructive pulmonary disease; *HR*, hazard ratio; *%FEV1*, percent predicted forced expiratory volume in one second; *%VC*, percent predicted vital capacity; *PNI*, prognostic nutritional index
